# *U2AF1* mutation promotes tumorigenicity through facilitating autophagy flux mediated by FOXO3a activation in myelodysplastic syndromes

**DOI:** 10.1038/s41419-021-03573-3

**Published:** 2021-06-28

**Authors:** Yuqian Zhu, Dandan Song, Juan Guo, Jiacheng Jin, Ying Tao, Zheng Zhang, Feng Xu, Qi He, Xiao Li, Chunkang Chang, Lingyun Wu

**Affiliations:** Department of Hematology, Shanghai Jiao Tong University Affiliated Sixth People’s Hospital, Shanghai, China

**Keywords:** Cell biology, Molecular biology

## Abstract

Mutations in the U2 small nuclear RNA auxiliary factor 1 (*U2AF1*) gene are the common feature of a major subset in myelodysplastic syndromes (MDS). However, the genetic landscape and molecular pathogenesis of oncogenic *U2AF1*^*S34F*^ mutation in MDS are not totally understood. We performed comprehensive analysis for prognostic significance of *U2AF1* mutations in acute myeloid leukemia (AML) cohort based on The Cancer Genome Atlas (TCGA) database. Functional analysis of *U2AF1*^*S34F*^ mutation was performed in vitro. Differentially expressed genes (DEGs) and significantly enriched pathways were identified by RNA sequencing. The forkhead box protein O3a (FOXO3a) was investigated to mediate the function of *U2AF1*^*S34F*^ mutation in cell models using lentivirus. Chromatin immunoprecipitation, immunoblotting analyses, and immunofluorescence assays were also conducted. *U2AF1* mutations were associated with poor prognosis in MDS and AML samples, which significantly inhibited cell proliferation and induced cellular apoptosis in cell models. Our data identified that *U2AF1*-mutant cell lines undergo FOXO3a-dependent apoptosis and NLRP3 inflammasome activation, which induces pyroptotic cell death. Particularly, an increase in the level of FOXO3a promoted the progression of MDS in association with restored autophagy program leading to NLRP3 inflammasome activation in response to *U2AF1*^*S34F*^ mutation. Based on the result that *U2AF1*^*S34F*^ mutation promoted the transcriptional activity of *Bim* through upregulating FOXO3a with transactivation of cell cycle regulators *p21*^*Cip1*^ and *p27*^*Kip1*^, FOXO3a, a potentially cancer-associated transcription factor, was identified as the key molecule on which these pathways converge. Overall, our studies provide new insights that *U2AF1*^*S34F*^ mutation functions the crucial roles in mediating MDS disease progression via FOXO3a activation, and demonstrate novel targets of *U2AF1* mutations to the pathogenesis of MDS.

## Introduction

Myelodysplastic syndromes (MDS) are clonal hematopoietic stem cell malignancies that are characterized by inefficient hematopoiesis, progressive bone marrow (BM) dysplasia, and increased mortality due to the progression to acute myeloid leukemia (AML) [1]. Peripheral blood cytopenia can be manifested by a large proportion of MDS sufferers despite normal or hypercellular BM. Excessive cell death and cell differentiation disorder in the early stage of MDS, and abnormal hematopoietic stem and progenitor cells (HSPCs) show apoptosis resistance when the disease progresses [2]. MDS consist of a heterogeneous group, which harbor a spectrum of chromosome abnormalities and somatic gene mutations. Previous studies have shown that MDS can be initiated by genetic or epigenetic modifications to HSPCs or their functions [3]. How genetic alterations trigger the diverse MDS phenotype remains unclear.

As parts of ribonucleoprotein complexes, spliceosomes proteins are associated with the splicing of introns when pre-mRNA matures. As a small subunit of U2AF, U2 small nuclear RNA auxiliary factor 1 (U2AF1) develops the U2AF heterodimer with a larger subunit U2AF2 through combination with the 3’ AG splice acceptor dinucleotide of the pre-mRNA target intron [4]. When analyzed in relation to other genes implicated in MDS, the compiled evidence shows that *U2AF1* is often the initial mutation that occurs [5, 6]. Although these studies have suggested that *U2AF1* mutations are selected early during tumorigenesis, mutations in a single allele of *U2AF1* that lead to a predilection for carcinogenesis and tumor progression are unexplained. The highly conserved serine at amino acid position 34 (S34F) is where U2AF1 mutations are commonly found [7]. The *U2AF1*^*S34F*^ mutation showed altered secretion patterns of interleukin 8 (IL-8) and IL-1α in MDS, supporting the hypothesis that the inflammatory response is a driver during cancer progression [8]. In addition, NLRP3 inflammasome-dependent IL-1β and IL-18 production is reported in S34F mutant *U2AF1*-expressing cells, which induces HSPCs pyroptosis, a caspase-1-dependent programmed cell death [9]. However, the specificity and regulatory mechanism of the *U2AF1*^*S34F*^ mutation in promoting NLRP3 inflammasome activation remain to be elucidated.

Here, we report that forkhead box protein O3a (FOXO3a), as a key transcription factor, is markedly increased in S34F mutant *U2AF1*-expressing cells. Moreover, our data strengthen the concept that the presence of a functionally active FOXO3a binding site for gene expression of cell cycle regulators induces consequent cell fate determination via FOXO3a-mediated autophagic flux and NLRP3 inflammasome activation, suggesting a potential therapeutic target in MDS and myeloid malignancies.

## Results

### Mutations of *U2AF1* are correlated with a poor prognosis

**Fig. 1:**
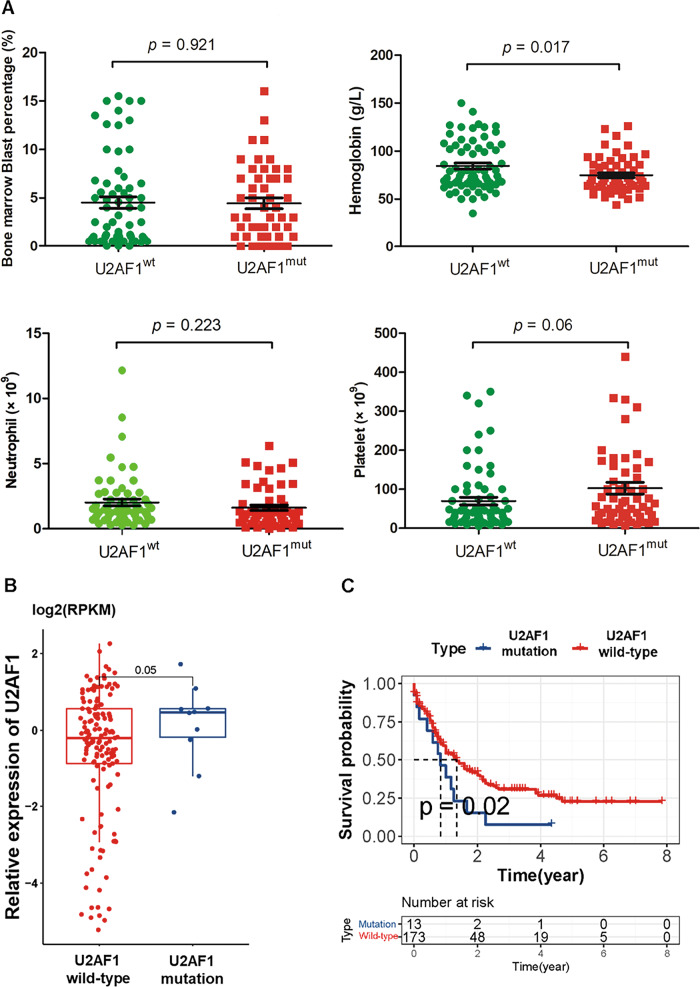
Mutations of U2AF1 in clinical MDS samples. **A** The patients carrying the *U2AF1* mutations (*n* = 58) revealed lower peripheral blood cell counts compared with those without a *U2AF1* mutation (*n* = 68). **B** Relative expression of *U2AF1* from TCGA-seq dataset. No significant differences were found for U2AF1 gene expression between mutant and wild-type samples (*p* > 0.05). **C** Kaplan–Meier survival analysis indicated that patients with *U2AF1* mutations were significantly correlated with a shorter overall survival than those without the mutation (*p* < 0.05). Statistical significance in relative expression analysis was determined by log2(RPKM) test.

Several sequencing studies have found that *U2AF1* is frequently mutated in MDS [10, 11]. Studies on the conditional knock-in alleles of mutant *U2af1* vivo models have significantly promoted the understanding of the pathogenesis of MDS despite several limitations in the presentation of clinical features [5]. Correlative analysis of *U2AF1* mutations with clinical features in MDS patients suggested that patients with *U2AF1* mutations had significantly lower hemoglobin percentages when compared with patients with wild-type *U2AF1* (*p* = 0.017; Fig. 1A). We previously evaluated the prognostic value of *U2AF1* mutations in MDS cohort samples, in which patients with *U2AF1* mutations were found to have lower survival than patients with wild-type *U2AF1* [10, 11]. The results shown in the box plots revealed that there was no difference in *U2AF1* expression between mutant and wild-type samples in The Cancer Genome Atlas (TCGA) database (*p* > 0.05; Fig. 1B). Consistently, the survival curves of AML patients revealed a trend that *U2AF1* mutations were significantly related to a shorter overall survival compared with those without a *U2AF1* mutation by TCGA (*p* = 0.02; Fig. 1C). Taken together, these clinical data strongly associate *U2AF1* mutations with a poor prognosis.

### U2AF1 mutation regulates the proliferation and apoptosis of SKM-1 and K562 cells

**Fig. 2:**
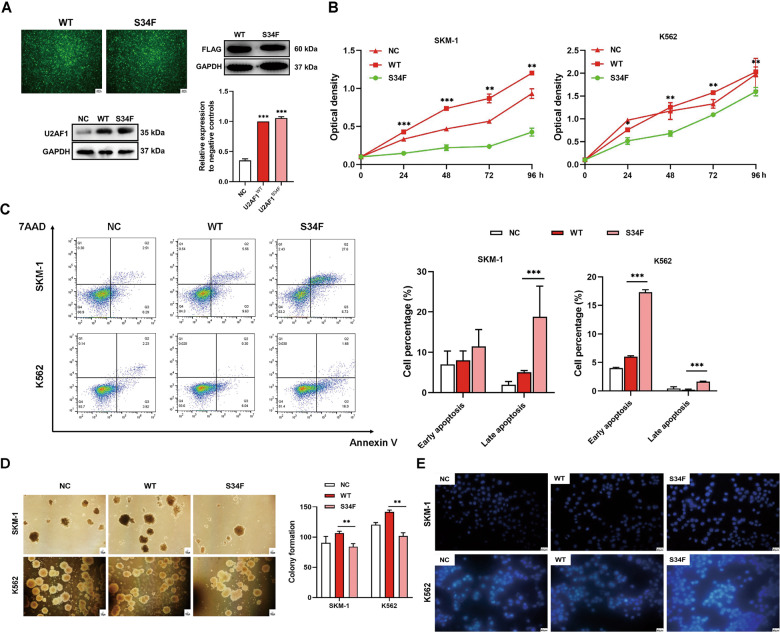
S34F mutant U2AF1 increases apoptosis and inhibits the viability of both K562 and SKM-1 cell lines. **A** Transfection efficiency under the fluorescence microscope. Scale bar, 50 µm. Western blotting analysis of FLAG-tagged wild-type or mutated protein level in stably transfected cells. Western blotting analysis to determine the protein expression levels of U2AF1^WT^ or U2AF1^S34F^ in stably transfected cells. **B** CCK-8 assays for investigating the proliferation capacity of WT and S34F mutant *U2AF1*. **C** Annexin V-APC/7-AAD double staining was utilized to detect cellular apoptosis by flow cytometry in both K562 and SKM-1 cell lines after wild-type and S34F mutant *U2AF1* treatment for 96 h. **D** Colony formation ability of wild-type and S34F mutant *U2AF1* in the both is shown; right histogram represents quantification analysis. Scale bar, 100 µm. **E** Hoechst 33258 staining of the cellular nuclei was performed to obverse apoptosis in both K562 and SKM-1 cells. Scale bar, 40 µm. The data are presented as the mean ± SD as well as the representative of no less than two single experimental processes. *p < 0.05, **p < 0.01, and ***p < 0.001. WT wild-type, NC negative controls.

Mutation screening indicated that wild-type *U2AF1* is expressed in K562 and SKM-1 cell lines (Supplementary Data 1). To determine whether the *U2AF1* mutant plays biological roles in MDS, we constructed a recombinant lentivirus with FLAG-tagged wild-type or *U2AF1* (S34F) mutant and stably transfected SKM-1 and K562 cells. The protein expression levels of U2AF1^S34F^ and U2AF1^WT^ compared with the levels in negative controls (NC) were confirmed by western blotting (Fig. 2A). The exogenous U2AF1^S34F^ and U2AF1^WT^ overexpression driven by lentiviral vectors, led to a significant increase of total U2AF1 protein levels in stably transduced cells. Nearly 95% of the cells expressing either FLAG-tagged wild-type or mutant U2AF1 were used for biological function studies. CCK-8 assays revealed that the *U2AF1* (S34F) mutant inhibited the viability of K562 and SKM-1 cells (Fig. 2B). Moreover, colony formation assays indicated that *U2AF1* (S34F) mutant-expressing cells exhibited significantly decreased proliferation capacity compared with cells expressing wild-type *U2AF1* in both cell lines (Fig. 2D). We also determined the effects of the *U2AF1* mutant on cellular apoptosis through Annexin V-APC/7-AAD double staining. Our data demonstrated a substantial increase in the proportion in SKM-1 in late apoptosis cells, and this trend was confirmed with K562 cells, which also showed a progressive increase in apoptosis rate (Fig. 2C). Then, *U2AF1-*mutant-induced apoptosis was further examined by Hoechst 33258 staining, with the results showing that the apoptotic portion constituted nearly three-fifths of the whole population (Fig. 2E). There were no significant differences between the cells expressing wild-type *U2AF1* and the negative control cells. Together with these results, we concluded that the *U2AF1*^*S34F*^ mutation could increase the apoptosis rate and suppressed cellular viability and colony formation in MDS and myeloid malignancies.

### *U2AF1* mutation affects gene expression profiles of SKM-1 cells

**Fig. 3:**
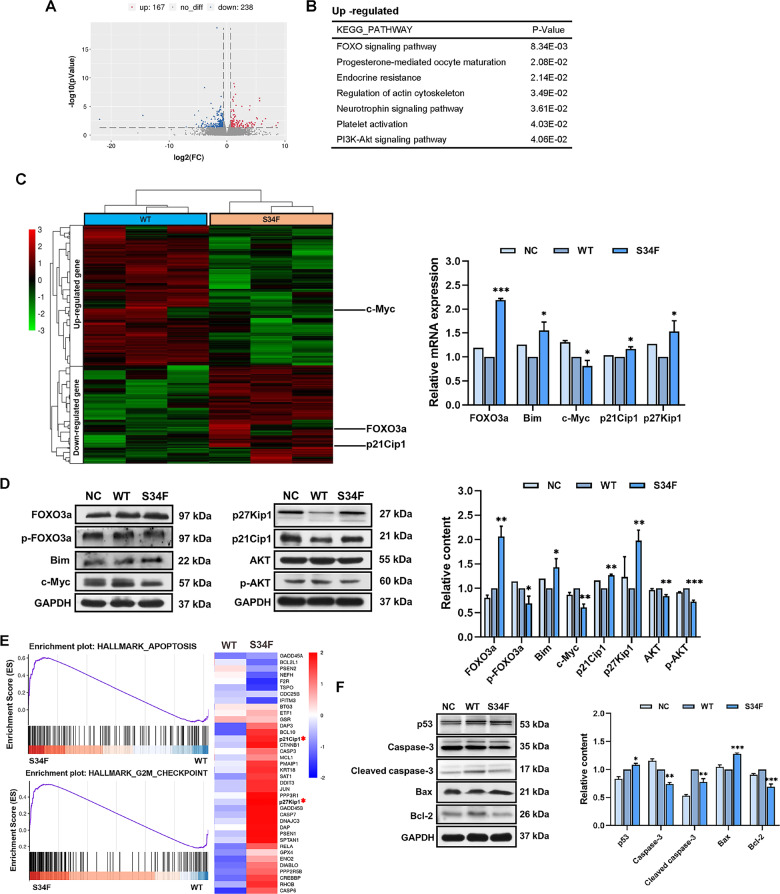
S34F mutant U2AF1 affects gene expression in SKM-1 cell line. **A** The volcano plot indicates the distribution of DEGs based on the RNA-seq data obtained from wild-type samples as well as S34F mutant *U2AF1* samples. **B** The pathway enrichment analysis on the upregulated DEGs of KEGG. **C** Hierarchical clustering plot of the fold changes (FC) of gene expressions in six samples induced by S34F mutant *U2AF1* detected by RNA-seq (FC > 1.5, *p*-values < 0.05). The mRNA expression of FOXO3a, Bim, c-Myc, p21^Cip1^, and p27^Kip1^ in SKM-1 cells treated with wild-type and S34F mutant *U2AF1* by qRT-PCR. **D** The altered protein levels of *U2AF1*^*S34F*^ mutation-associated genes were validated by western blotting in SKM-1 cells. Quantitative analysis of western blotting bands from three independent experiments is shown. **E** Heatmap showing the enriched genes in the apoptosis pathway of GSEA results between wild-type and S34F mutant *U2AF1*-treated SKM-1 cells (FDR q < 0.25). **F** Representative bands of western blotting data showing the protein levels of p53, Cleaved caspase-3, caspase-3, Bcl-2 as well as Bax in different groups based on the GSEA results. The data represent at least two independent experiments with three samples in each group. *p < 0.05, **p < 0.01, and ***p < 0.001. WT wild-type, NC negative controls.

To understand how the cancer-associated *U2AF1*^*S34F*^ mutation promotes MDS, we next compared independent RNA samples of SKM-1 cells expressing wild-type *U2AF1* and S34F mutant *U2AF1* using RNA sequencing (RNA-seq). A volcano plot is presented to show the expression profiles (Fig. 3A). Our RNA-seq data revealed 405 differentially expressed genes (DEGs), including 167 upregulated and 238 downregulated genes (fold changes (FC) cutoff of 1.5; *P* < 0.05). Through RNA-seq of three pairs of independent RNA samples from *U2AF1*-mutant and *U2AF1*-WT cell lines, we determined the transcriptome landscape and identified the potential core genes based on hierarchical clustering analysis (Fig. 3C). Among the 405 DEGs, *FOXO3a* (FC = 1.64 and *P* = 2.35E-02) and *CDKN1A* (*p21*^*Cip1*^, FC = 1.63, and *P* = 1.80E-02) were significantly upregulated, while *c-Myc* (FC = 0.09, *P* = 8.70E-04) was notably downregulated. These genes were further verified by qRT-PCR. Consistently, the relative mRNA expression levels of *FOXO3a* and *p21*^*Cip1*^ showed a significantly increasing trend in the *U2AF1* (S34F) mutant-transduced SKM-1 cells. In line with the mRNA expression data, *c-Myc* was transcriptionally inactivated following the overexpression of the *U2AF1* mutant.

We then sought to determine whether the expression of the downstream targets cooperates with FOXO3a activation during the tumorigenesis of *U2AF1*-mutant-expressing cells. Western blotting results revealed that the *U2AF1*^*S34F*^ mutation was associated with substantially increased protein levels of FOXO3a, BCL2L11 (Bim), p21^Cip1^, and CDKN1B (p27^Kip1^) compared with the two controls, whereas the levels of phosphorylated FOXO3a (p-FOXO3a) and c-Myc were decreased (Fig. 3D). The results of Kyoto Encyclopedia of Genes and Genomes (KEGG) pathway enrichment analysis showed that these DEGs were involved in several biological pathways, including the forkhead box protein O (FOXO) and PI3K/Akt signaling pathways (Fig. 3B). Consistently, the phosphorylation levels of AKT were significantly attenuated in the *U2AF1* (S34F) mutant group. Conducting GSEA of the RNA-seq data illustrated that genes related to G2/M checkpoint and apoptosis-associated pathways were statistically enriched in *U2AF1* (S34F) mutant-expressing cells (FDR *q* < 0.25; Fig. 3E). As shown in Fig. 2F, the western blotting analyses also demonstrated altered protein levels, specifically increased expression of p53 and Bax and decreased protein levels of Bcl-2 and cleaved caspase-3 in *U2AF1* (S34F) mutant-expressing cells, indicating that the *U2AF1*^*S34F*^ mutation-induced apoptosis could be mediated by Bcl-2 family genes.

### *U2AF1* mutation induces G2/M cell cycle arrest mediated by FOXO3a activation

**Fig. 4:**
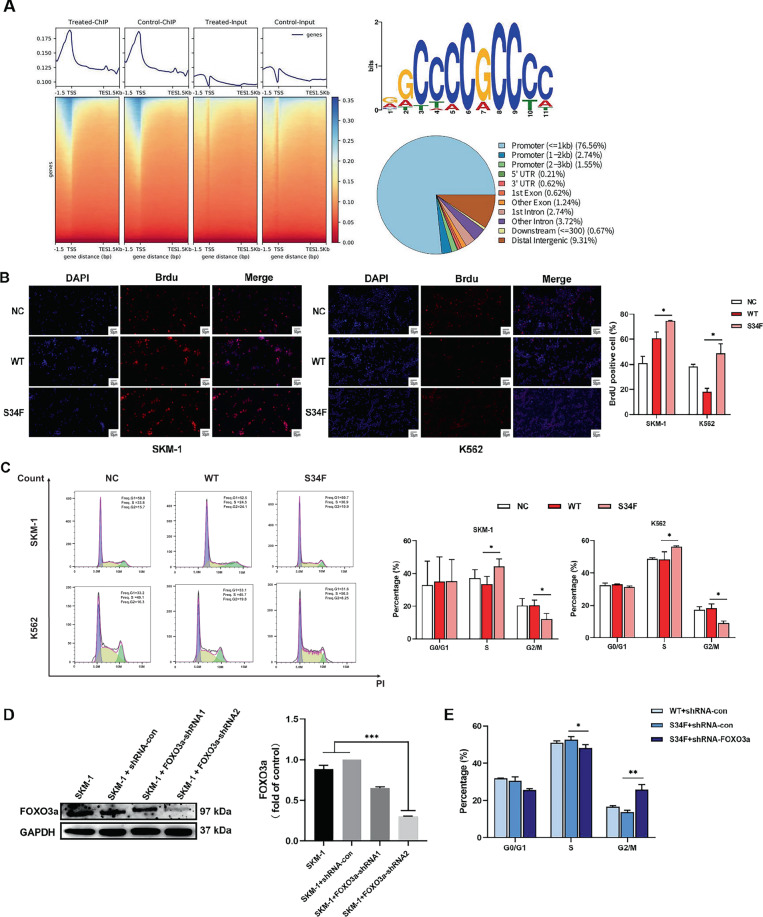
S34F mutant U2AF1 induces G2/M cell cycle arrest mediated by FOXO3a activation. **A** ChIP-seq for FOXO3a binding to the promoter regions. Heatmaps and average intensity curves of ChIP assay reads for FOXO3a. **B** The representative micrographs and quantification of BrdU-incorporating cells (red) in both K562 and SKM-1 cell lines are shown. Nuclei counterstained with DAPI. Scale bar, 50 µm. **C** The representative flow cytometry analysis of the cell cycle distribution after efficient transfection for 96 h in both K562 and SKM-1 cells. S34F mutant *U2AF1* induced the increase in the proportion of S-phase cells and significantly reduced the percentage of cells at G2/M phase. **D** The knockout efficiency for FOXO3a was determined by western blotting assays in SKM-1 cells. **E** Gene silencing of FOXO3a rescued the G2/M blocks induced by S34F mutant *U2AF1* in SKM-1 cells. The data represent all experiments in triplicate for each cell line. *p < 0.05, **p < 0.01, and ***p < 0.001. WT wild-type, NC negative controls.

It has been shown that tumorigenesis is promoted by the upregulation of FOXO3a in AML [12]. We next performed chromatin immunoprecipitation sequencing (ChIP-seq) with FOXO3a to analyze the effect of mutant *U2AF1* on the genetic landscapes and chromatin states in MDS. According to the ChIP assays, FOXO3a specifically binds to a site in the proximal promoter region. In addition, our enhancer analysis indicated that the *U2AF1* (S34F) mutant increased the average FOXO3a level at the transcription start site (Fig. 4A). Recent studies have also revealed that FOXO3a could regulate *Bim* transcription through binding its promoter regions, mediating cell cycle distribution [13]. Above data highlighted the cell cycle regulators, *p21*^*Cip1*^ and *p27*^*Kip1*^, were transcriptionally activated following *U2AF1* (S34F) mutant expression, and this effect was accompanied by upregulated FOXO3a and downregulated c-Myc expression levels. In an effort to discover the underlying molecular mechanism of *U2AF1* (S34F) mutant in conjunction with an increase in the expression levels of cell cycle regulators, we performed 5-bromo-2’-deoxyuridine (BrdU) incorporation assays with SKM-1 and K562 cell lines. As shown in Fig. 4B, the expression of the *U2AF1* (S34F) mutant significantly increased the proportion of BrdU-positive cells and the percentage of cells in the S-phase and significantly reduced the percentage of cells in the G2/M phase, which suggested the induction of cell cycle arrest by blocking the S/G2 checkpoint in both cell lines (Fig. 4C). Ninety-six hours after transduction with the FOXO3a-shRNA lentiviral particles, the protein expression levels of FOXO3a were significantly downregulated (Fig. 4D). Cell cycle analyses revealed that the downregulation of FOXO3a significantly attenuated a considerable portion of the *U2AF1* (S34F) mutant-induced cells in S-phase and partly reversed the G2/M blockades, indicating that FOXO3a plays an important role in *U2AF1* (S34F) mutant-induced SKM-1 cell cycle arrest (Fig. 4E).

### Activation of the NLRP3 inflammasome in *U2AF1*-mutant-expressing cells by FOXO3a

**Fig. 5:**
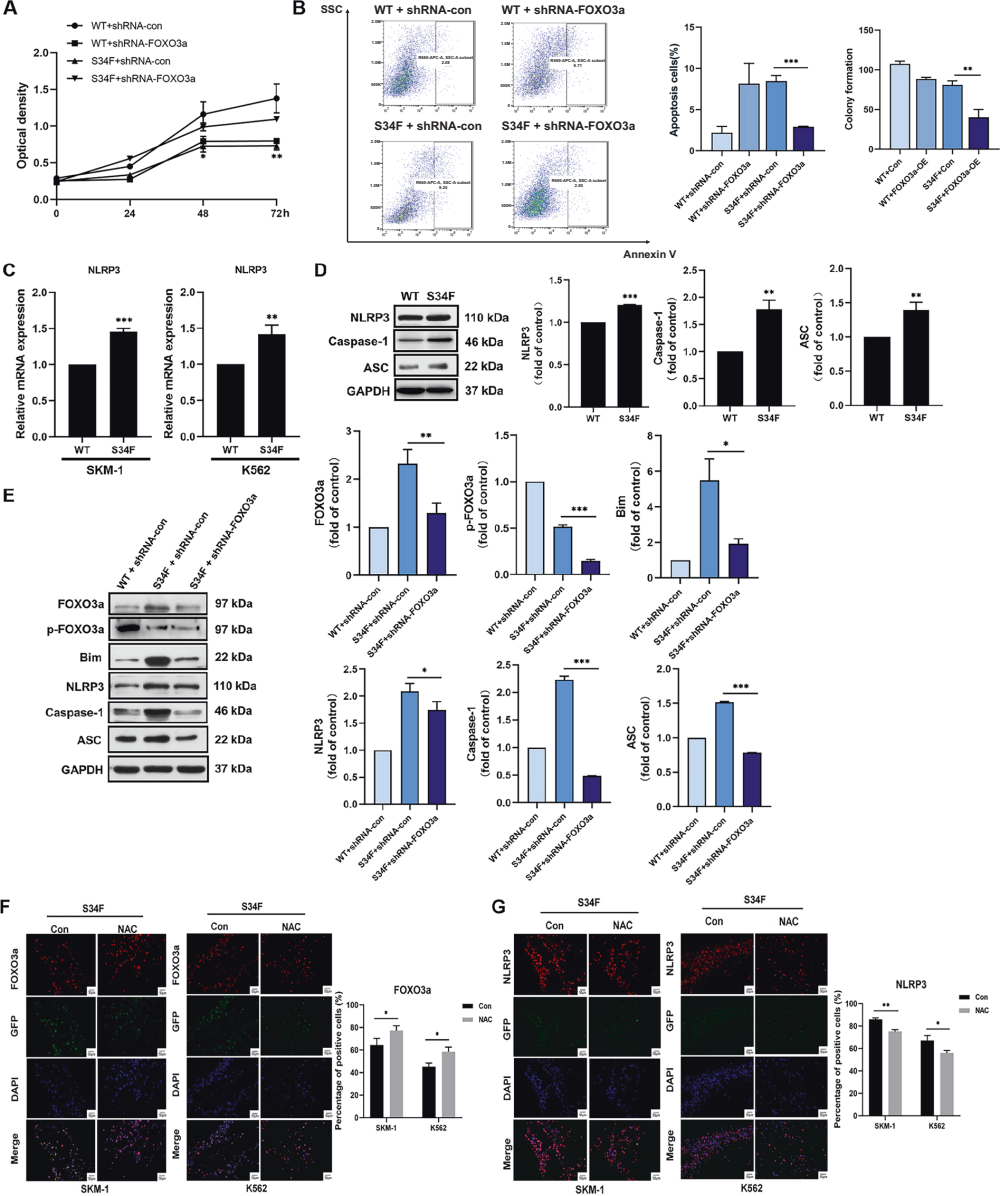
Pyroptosis of S34F mutant U2AF1-expressing cells as induced by FOXO3a-mediated NLRP3 inflammasome activation. **A** The proliferation curve of SKM-1 cells superinfected by FoxO3a-shRNA lentiviral particles at the specified time point. Then silencing of FOXO3a overcame the suppressive growth of S34F mutant *U2AF1*. **B** FOXO3a suppression abolished the induction of cellular apoptosis in S34F mutant *U2AF1*-expressing cells by flow cytometry using Annexin V single-staining. Overexpression of FOXO3a (FOXO3a-OE) rescued the growth capacity induced by S34F-shRNA-FoxO3a in SKM-1 cells. **C, D** Western blotting or qRT-PCR assays for NLRP3 inflammasome or other inflammatory biomarkers in SKM-1 or K562 cells treated with wild-type and S34F mutant *U2AF1* for 96 h. **E** Western blotting analysis for FOXO3a and NLRP3 inflammasome markers in SKM-1 cells expressing wild-type *U2AF1* and S34F mutant *U2AF1* after transfection with the FOXO3a-shRNA lentiviral particles for 96 h. Band intensities represent values relative to control group. **F, G** Representative photomicrographs showed cells expressing S34F mutant *U2AF1* containing green fluorescence protein (green) immunolabeled using the anti-FOXO3a and anti-NLRP3 antibody (red). Nuclei were stained with DAPI (blue). Scale bar, 50 µm. Magnification, ×200. Upper histogram represents quantification analysis. The data are presented as the means ± SD of multiple experiments conducted in triplicate. *p < 0.05, *p < 0.01, and ***p < 0.001. WT wild-type.

Because the altered expression of FOXO3a has been reported to influence SKM-1 cell growth [13], the effects of FoxO3a on biological functions of S34F mutant *U2AF1*-expressing cells were determined. As shown in Fig. 5A, the *U2AF1* (S34F) mutant-positive group with shRNA- FOXO3a-expressing cells exhibited a higher proliferation rate than the group overexpressing only the *U2AF1* mutant. To further investigate the effects of *U2AF1*^*S34F*^ mutation in cell models, the apoptotic ratio was detected by Annexin V single-labeling of specific cells. Silencing FOXO3a reduced the population of Annexin V-positive cells as mediated by the *U2AF1* (S34F) mutant (Fig. 5B). Consistently, colony formation assays revealed that *U2AF1* (S34F) mutant-expressing cells transfected with the FOXO3a overexpression plasmid (FOXO3a-OE) showed significantly attenuated the proliferation capacity. These results confirm that the regulatory effect of *U2AF1*^*S34F*^ mutation on cell proliferation and apoptosis was significantly reversed by silencing of FOXO3a.

Previous studies have illustrated that FOXO3a is critically involved in the inflammatory response mediated by the NLRP3 inflammasome in the malignant progression of tumors [14, 15]. However, the role of FOXO3a in MDS disease progression has not been reported so far. Here, we focused on the relationship between FoxO3a overexpression and NLRP3 inflammasome in *U2AF1* (S34F) mutant-expressing cells. As shown in Fig. 5C, the *NLRP3* mRNA levels were upregulated in both cells compared with the controls. The *U2AF1* (S34F) mutant increased NLRP3 expression, and the ASC and activated caspase-1 levels were also upregulated (Fig. 5D). Moreover, western blotting analysis indicated that FOXO3a silencing significantly decreased the levels of NLRP3, ASC and active caspase-1 that were triggered by S34F mutant *U2AF1* (Fig. 5E). Additionally, the expression level of Bim, an apoptosis-related FOXO3a downstream target protein, was decreased significantly after coinfection. Reactive oxygen species (ROS) is critical for promoting the formation of NLRP3 inflammasome [9]. To further clarify the localization of FOXO3a and NLRP3, we abrogated ROS in SKM-1 and K562 cells by antioxidant N-acetylcysteine (NAC) treatment. As indicated in Fig. 5F, G, the immunostaining results using an anti-NLRP3 antibody showed that NAC effectively reduced NLRP3 inflammasome activation in the *U2AF1* (S34F) mutant cells. By contrast, the number of FOXO3a-positive cells was significantly increased after treatment with the antioxidant NAC compared with that of the controls. These findings in aggregate indicate that S34F mutant *U2AF1* affects NLRP3 inflammasome activation by regulating FOXO3a signaling, dependently of its apoptotic activity.

### Mutant *U2AF1* restores autophagy flux as a result of FOXO3a dysregulation

**Fig. 6:**
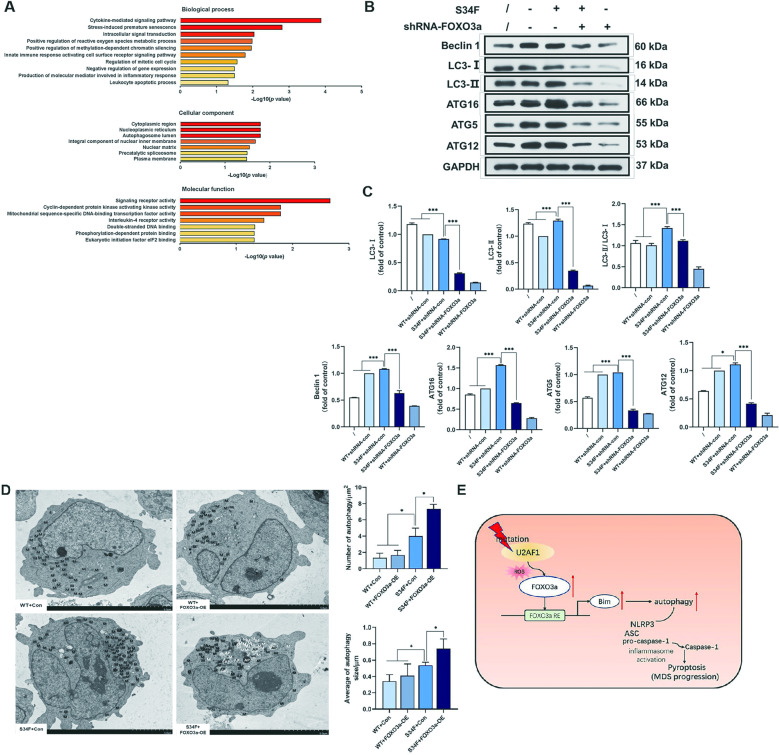
Restored autophagy program as a result of FOXO3a dysregulation by U2AF1S34F mutation. **A** Biological process, molecular function, as well as cellular component are adopted to categorize GO. **B, C** Representative images of western blotting data. Quantitative analysis of the protein expression levels, and the LC3-II/ LC3-I ratio is demonstrated. Beclin 1, ATG5, ATG12, and ATG16 complex proteins were all up-regulated following S34F mutant *U2AF1* treatment. These proteins were decreased after silencing of FOXO3a. **D** Representative transmission electron micrographs of autophagosomes ultrastructure. Scale bar, 5 µm. **E** Schematic representation of the potential role of *U2AF1*^*S34F*^ mutation based on this study. The data represent at least two independent experiments with three samples per group in each. *p < 0.05, **p < 0.01, and ***p < 0.001. WT, wild-type, M mitochondria, AP autophagosome.

Previous results indicated that FOXO3a contributed to signaling pathways mediating autophagy and inflammatory responses [13]. Since the impaired autophagy program leads to NLRP3 activation [16], we wondered whether FOXO3a was capable of regulating autophagy flux in the *U2AF1* (S34F) mutant cells. Gene Ontology (GO) analysis made us able to identify molecules associated with the biological process of positive regulation of reactive oxygen species-associated metabolic process and the cellular component of autophagosome lumen (Fig. 6A). We next determined the levels of autophagy-related genes by western blotting analyses. As shown in Fig. 6B, the levels of the autophagy-initiating proteins Beclin 1, ATG5, ATG12, and ATG16, as well as anti-light chain 3 (LC3)-I and LC3-II, in both the S34F-shRNA-FOXO3a and WT-shRNA-FOXO3a groups were decreased compared with those in the WT-shRNA-con group, but an increase was observed in the S34F group. The transformation of the nonlipidated LC3 form, LC3-I, to the lipidated form, LC3-II, is a general marker of autophagic activity [13, 17]. The LC3-II/LC3-I ratio in the *U2AF1* (S34F) mutant-expressing cells was significantly higher than that in the WT-shRNA-con group, which is indicative of autophagy induction. Following FOXO3a silencing, the levels of both LC3-I and LC3-II were attenuated, as well as the ratio of LC3-I and LC3-II (Fig. 6C). Similar results were obtained by transmission electron microscopy of the SKM-1 cells overexpressing FOXO3a. Morphological changes in autophagosomes were observed by transmission electron micrographs (Fig. 6D). Ultrastructural analysis suggested an increase in the number and size of autophagosomes in cells expressing S34F mutant *U2AF1*, which was eventually enhanced by the addition of FOXO3a, indicating that FOXO3a is closely associated with oxidative stress by regulating autophagic flux. Overall, the above results demonstrate that the effects of autophagy flux exerted by the *U2AF1*^*S34F*^ mutation are achieved through FOXO3a activation.

## Discussion

Mutant *U2AF1*, located in the highly conserved zinc fingers, facilitates increased splicing and exon skipping, disrupting the capability of U2AF1 to RNA splicing machinery [18, 19]. Abnormalities in spliceosome genes are often the initial mutation, while the remaining genetic aberrations appear gradually during the evolution of MDS [5, 6]. Nevertheless, only a single copy of the gene is mutated in malignancies, proposing the possibility that the mutations do not lead to a simple loss of function.

Because of the complexity of its cytogenetic pathogenesis, the prognostic effect of the *U2AF1*^*S34F*^ mutation in MDS remains controversial. The present study reveals that the *U2AF1*^*S34F*^ mutation is significantly associated with poor prognosis in patients with AML based on the TCGA database. Consistently, we previously found that patients with MDS harboring *U2AF1* mutations had a shorter overall survival compared with those without a *U2AF1* mutation, and patients with *U2AF1* mutations usually had a higher risk of AML transformation [10]. Different phenotypes have been observed in vitro models of primary human BM CD34+ progenitor cells. *U2AF1*^*S34F*^ mutation in human HSPCs impaired erythroid differentiation and led to the skewing of granulomonocytic differentiation towards granulocytes [20]. A defining feature of lower-risk MDS is BM failure, and excessive HSPCs apoptosis contributes toward the ineffective hematopoiesis characteristic of MDS [2]. In our study, we demonstrated that *U2AF1* mutations in patients with MDS were related to lower hemoglobin percentages when compared with patients with wild-type *U2AF1*. Moreover, our biological assays showed that the *U2AF1* (S34F) mutant suppressed K562 and SKM-1 cell proliferation by strongly inducing apoptosis and a G2/M phase blockade. How the *U2AF1*^*S34F*^ mutation confers a clonal growth advantage in MDS is unclear.

Here, we used RNA-seq to identify 405 DEGs and found that biological processes were enriched in cytokine-mediated and FOXO signaling pathways. Among these DEGs, *FOXO3a* and its target molecule *p21*^*Cip1*^ were observed to have higher expression in the mutant cells than that in controls, as well as reduced expression of *c-Myc*. FOXO3a functions as a potential oncoprotein by upregulating molecules involved in the cell cycle, apoptosis, and autophagy [13]. To explore the potential regulatory mechanisms involving the FOXO signaling pathway, FOXO3a was knocked out using SKM-1 cells superinfected with FOXO3a-shRNA lentiviral particles. Our data demonstrated that the decrease in FoxO3a expression overcame the tendency toward an increased ratio of apoptotic cells as mediated by the *U2AF1*^*S34F*^ mutation.

The FOXO transcription factor family contains four highly relevant members, namely, FOXO1, FOXO3a, FOXO4, and FOXO6, which are direct downstream targets of AKT and play important roles in cancer progression [12, 21]. Previously, it has been shown that FOXO1 belongs to positive transcription factor involved in the stress response pathway [14]. In a Gene Expression Omnibus (GEO) dataset (GSE19429) analysis of CD34+ cells of patients with MDS, *FOXO1* was found to be significantly downregulated [22, 23]. Moreover, PPI network construction using STRING tools indicated *FOXO1* as a core gene interacting with other genes that also have roles in apoptosis. According to RNA-seq data, apoptosis pathways were dramatically affected by *the U2AF1*^*S34F*^ mutation, including the level of *Bim*, which is a proapoptotic factor in AML cells [24]. It has been reported that activated FOXO3a regulates the transcription of *Bim* gene by binding to the *Bim* promoter [25, 26]. Combining RNA-seq and ChIP-seq data, we demonstrated that FOXO3a elevation is a transcription inducer of FOXO3a-Bim axis genes in the *U2AF1* (S34F) mutant samples, consistent with a previously predicted motif [27]. These results support the conclusion that FOXO3a has a strong positive impact on enhancer states and that Bim is a crucial downstream regulator of *U2AF1*^*S34F*^ mutation-induced abnormalities in apoptosis.

Another intriguing discovery of our research is the finding that overexpression of FOXO3a mediated by the *U2AF1* (S34F) mutant promotes NLRP3 inflammasome activation in SKM-1 cells. Full formation of the NLRP3 inflammasome then induces IL-1β release and caspase-1 production, hallmarks of pyroptosis [9]. Knocking down FOXO3a lowered NLRP3 activity, ASC, and active caspase-1 levels in the present study. Autophagy is a highly conserved self-digestion process that is an essential mechanism for maintaining metabolic homeostasis and energy balance in cells [17, 28]. Given the importance of NLRP3-dependent inflammatory cell death in impairing HSPCs survival in MDS, previous studies reported that impaired autophagy led to NLRP3 activation [16]. Park et al. have asserted that the *U2AF1*^*S34F*^ mutation promoted ATG7 alternate splicing, which disrupted autophagy program [29]. Interestingly, our results showed that the autophagy flux previously induced by the *U2AF1* (S34F) mutant can be partially attenuated by silencing FOXO3a. FOXO3a participates in the regulation of autophagy by inducing the expression of downstream target genes, such as Bim, p21^Cip1^, p27^Kip1^, and FasL [30]. These genes are also involved in regulating apoptosis, autophagy and ROS. In our studies, S34F mutant *U2AF1* promoted the transcriptional activity of *Bim* through upregulating FOXO3a by depressed PI3K/AKT activity accompanied by transactivation of cell cycle regulators *p21*^*Cip1*^ and *p27*^*Kip1*^, as well as decreased *c-Myc* expression, leading to the activation of autophagic flux.

It is well established that tumor-promoting inflammation and genome instability are considered as the pathogenic hallmarks of cancer [31]. FOXO3a, a potentially cancer-associated transcription factor, is involved in the intersection of multiple signal pathways of oxidative stress, which causes a cascade of pathological processes by activating diverse cellular processes, including apoptosis, autophagy and immune-inflammatory responses [13]. However, the specific contribution of FOXO3a in S34F mutant *U2AF1*-expressing cells to MDS disease progression and the associated molecular mechanisms were not yet fully understood. Our result that FOXO3a enhanced programmed cell death activity fortifies the effect of *U2AF1*^*S34F*^ mutation on inflammasome activation in MDS. NADPH oxidase (NOX) facilitates the production of ROS, which activates the NLRP3 inflammasome pathway when the *U2AF1*^*S34F*^ mutation is expressed [9, 32]. Nevertheless, our data showing that FOXO3a activity remained elevated after treatment with the antioxidant NAC further supports the direct effect of FOXO3a on NLRP3 inflammasome activation in the *U2AF1*-mutant cell lines. More importantly, as the early events, the phenotypic changes produced by *U2AF1*^*S34F*^ mutation might indicate the initiation of downstream oncogene mutations and diverse signaling pathways affected in the multistep development of tumor inflammatory microenvironment, and coordinately cause AML transformation [32]. Elevated ROS levels lead to increased DNA damage, which could release related inflammatory cytokines by activating the NF-κB, Wnt/β-catenin and other inflammatory response pathways, and then form a vicious cycle with the release of persistent inflammatory factors, leading to the loss of tumor suppressor factors through somatic gene mutations and chromosome rearrangement. The results are in line with these previous studies showing that the *U2AF1*^*S34F*^ mutation is overrepresented in individuals with del (20q) and trisomy 8 [10, 18]. Although some studies show that FOXO3a acts as a tumor suppressor in cancer, we suggest that FOXO3a may play an important role in detrimental exaggeration, possibly related to the disease model. Therefore, future studies are needed to explore the role of FOXO3a in regulating MDS disease progression in vivo models.

In conclusion, our findings highlighted the functional importance of the *U2AF1*^*S34F*^ mutation in mediating MDS disease progression by activating the NLRP3 inflammasome induced by FOXO3a-mediated autophagic flux. The identification of FOXO3a as a regulator of pyroptosis, along with the transactivating effect of FOXO3a on gene expression of cell cycle regulators, sheds light on new avenues and potential prognostic biomarkers for the therapy of patients with MDS.

## Material and methods

### Patients and samples

A total of 126 patients diagnosed with MDS at our department from 2018 to 2020 were enrolled in this study. Diagnosis of MDS was based on the World Health Organization (WHO) criteria [33] and the minimum diagnostic standards [34] for MDS. This research was approved by the ethics committee of the Shanghai Jiao Tong University Affiliated Sixth People’s Hospital, and informed consent was obtained from all patients for this research in accordance with the Declaration of Helsinki.

### Cell line and culture

RPMI 1640 medium (Gibco, US) containing 10% heat-inactivated fetal bovine serum (Gibco, US), 100 μg/ml streptomycin, and 100 IU/ml penicillin was used to routinely culture the chronic myelogenous leukemia (CML) cell line K562 and the MDS-derived AML cell line SKM-1 (Health Science Research Resources Bank, Japan). Both K562 and SKM-1 cells were maintained at 37 °C under 5% CO_2_. In addition, the cell samples were recently authenticated by Short Tandem Repeat (STR) and tested for mycoplasma contamination.

### Reagents and antibodies

N-acetylcysteine was obtained from Selleck (S1623). Cell Signaling Technology (Danvers, MA, USA) provided antibodies against FOXO3a (#2497), phospho-FOXO3a (#13129), BCL2L11 (#2819), CDKN1A (#2947), CDKN1B (#3686), and c-Myc (#5605). Affinity Biosciences provided the anti-GAPDH antibody (AF7021). Abcam (Cambridge, MA, USA) provided anti-FLAG (ab205606), anti-U2AF1 (ab197591), anti-NLRP3 (ab210491), anti-Caspase-1 (ab207802), anti-ASC (ab151700), anti-AKT (ab32505), anti-phospho-AKT (ab192623), anti-Bcl-2 (ab182858), anti-Bax (ab32503), and anti-Cleaved Caspase-3 (ab32042) antibodies. Autophagy Antibody Sampler Kit (#4445) was obtained from Cell Signaling Technology (Danvers, MA, USA). Sangon Biotech (Shanghai, China) provided HRP-conjugated secondary antibodies.

### lentivirus infection assay

For cell transfection, wild-type and S34F mutant *U2AF1* were introduced into the GV492 (Ubi-MCS-3FLAG-CBh-gcGFP-IRES-puromycin) lentiviral vectors with the open reading frame (ORF) clone of human *U2AF1* (ref. ID NM_006758) and a carboxy-terminal FLAG-tag. Based on direct sequencing, this study identified the constructs overall. Plasmids were transfected with HitransG A reagent (GeneChem, Shanghai, China) at a corresponding MOI reaching 20 in accordance with the vendor’s directions when the cells reached 80% confluence. Cell samples were harvested 72 h after transfection, and in this study, stably transfected clones were transfected with 4 μg/ml puromycin and counted to determine the efficiency of green fluorescence protein (GFP). Normally, more than 80% of the cells were GFP-positive cells. By performing the western blotting assay and the flow cytometry analysis, we confirmed the transgene expression. Untransfected cells acted as negative controls. FoxO3a-shRNA (shRNA-FOXO3a) contained specific sequences targeting the *FOXO3a* sequence (5’-TTCCAAACTTGTACGCAGTTT-3’; 5’-AAGCTTGTCACTCCTGTTAAT-3’), and a control shRNA acted as a silencing negative control, as supplied by GeneChem Company (Shanghai, China). Cells were transfected following the manufacturer’s directions.

### Quantitative real-time PCR (qRT-PCR)

In accordance with the producer’s direction, the total cellular RNA was extracted with the EZ-Press RNA purification tool based on a spin column. RNA under isolation was reverse transcribed to obtain cDNA by complying with the manufacturer’s directions. Using an ABI 7500 real-time PCR machine (Applied Biosystems, Foster, CA, USA), qRT-PCR was performed based on Real Master Mix (TaKaRa, Dalian, China). GAPDH was selected as the endogenous control gene. With the 2^-△△Ct^ approach, the present study obtained relative expression levels of genes as fold variations. Supplementary Table S1 lists the primer sequences.

### Cell cycle analysis

Cell cycle distribution was analyzed utilizing cell cycle staining solution (Beijing Solarbio Science & Technology Co., Ltd) following the vendor’s protocol. The transfected cell samples were cleaned based on cold PBS, and cold 70% ethanol was introduced in a gentle and dropwise manner to fix the samples. Cells then received the pelleting and resuspending processes in propidium iodide staining solution (50 μg/ml RNase A and 50 μg/ml propidium iodide solution), followed by incubation at 37 °C under darkness for 30 min before flow cytometry analysis. FlowJo software was used to analyze the cell cycle.

### Apoptosis assay

For analysis of cellular apoptosis, the transfected cells were stained using an APC Annexin V Apoptosis Detection Kit with 7-AAD following the manufacturer’s directions. The stained cells were detected via flow cytometric analysis; all data were analyzed with FlowJo software.

### Cell proliferation assay

With Cell Counting Kit-8 (CCK-8; Dojindo, Kumamoto, Japan) assays, our study determined the cellular viability following the manufacturer’s directions. In summary, overall 5000 cells per well received the culturing process based on 96-well plates under 100 μl volume finally. Four parallel wells were seeded with the respective group of cell samples. The cell samples underwent a 4-day incubation in CO_2_ at 37 °C. At different time points, with the use of a microplate reading element (Thermo Scientific), 10 μl of CCK-8 solution was introduced in the respective well to measure absorbance at 450 nm. The optical density (OD) values of each well represented the viability of cells.

### Colony formation assay

Colony forming tests were conducted with 500 cells per well in six-well plates. The transfected cells were resuspended in MethoCult H4434 methylcellulose medium (StemCell Technologies) with granulocyte-macrophage colony-stimulating factor (GM-CSF), stem cell factor (SCF), erythropoietin (EPO), and interleukin 3 (IL-3) and plated in triplicate wells. The cell samples were incubated for fourteen days at 37 °C to allow colony formation, and colonies covering no less than 30 cells were scored after plating.

### RNA sequencing and analysis of integrative network

Cells were administered the vehicle control and S34F mutants in triplicate for 96 h, and total RNA was extracted with TRIzol reagent (Invitrogen, CA, USA) following the vendor’s process. RNA sequencing was performed on the Illumina X10 system of LC Sciences (USA). R software package was adopted for processing the DEGs. Pathway enrichment analyses of the dysregulated genes were also carried out. Our study employed the Gene Set Enrichment Analysis (GSEA) online database to identify the interactions of DEGs in the apoptosis-related cluster.

### Chromatin immunoprecipitation assay

Cells were harvested, and formaldehyde was introduced to the cell samples to a final concentration of 1% for crosslinking of chromatin for 10 min at ambient temperature. Subsequently, with 125 mM glycine, formaldehyde was inactivated. Chromatin digested into fragments of 100–300 bp received preclearing and subsequently immunoprecipitation with Protein A + G magnetic beads in combination with anti-FoxO3a antibodies (ab12162, Abcam). Based on the manufacturer’s directions, high-throughput ChIP fragment sequencing was conducted with Illumina HiSeq.

### Immunofluorescence assay

Cells were seeded on glass coverslips and fixed at ambient temperature for 15 min utilizing 4% paraformaldehyde. These cells were stained with anti-FOXO3a (1:200) and anti-NLRP3 (1:200) rabbit Abs and then incubated at 4 °C overnight. Next, the cell samples were incubated in fluorochrome-conjugated secondary antibodies at a 1:800 dilution for 1 h at ambient temperature under the darkness. After being cleaned, cells received the staining process with DAPI for 10 min at 37 °C. Six random immunostaining images of the specimens were captured under a fluorescence microscope; then, data were analyzed with Image-Pro Plus 6.0 software.

### Western blotting

The cultured cells were harvested and then lysed for 30 min on ice in RIPA (Beyotime, China). Next, cellular protein extracts were collected with centrifugation. Twenty five micrograms of proteins were resolved under SDS-PAGE and transferred to PVDF films. In 5% fat-free milk TBST solution, films received the blocking for 1 h and then the incubation throughout the night based on each major antibody at 4 °C. After being cleaned using TBST buffer, the films were incubated for 1 h at ambient temperature with the appropriate secondary antibodies. The proteins were visualized under improved chemiluminescence (ECL). Our study confirmed the equivalent loading of samples based on immunoblotting for GAPDH. Data were analyzed using ImageJ software, and figures were cropped.

### Statistical analysis

Data were presented as mean ± standard deviation (SD) based on SPSS statistics software version 23.0 from no less than two single experimental processes. Student’s *t*-test was used for the comparison of two conditions. One-way analysis of variance (ANOVA) was conducted for comparison of multiple conditions. Statistical significance was defined as *p < 0.05, **p < 0.01, and ***p < 0.001.

## Supplementary information

Supplementary information

Supplementary Data 1

## Data Availability

All data analyzed in this research are available from the correspondence on reasonable request. The expression profiling data have been deposited into the NCBI Gene Expression Omnibus under GEO Accession Number GSE166798.
