# Supplementary material for: U2AF1 mutation promotes tumorigenicity through facilitating autophagy flux mediated by FOXO3a activation in myelodysplastic syndromes
Source: Cell Death Dis. 2021 Jun 28;12(7):655. doi: 10.1038/s41419-021-03573-3 (PMC8238956; doi:10.1038/s41419-021-03573-3)
Supplement: Supplementary file 1 — Supplementary information [file 41419_2021_3573_MOESM1_ESM.docx]

**Supplementary Information**

**Supplemental Table S1.** Sequences of primers for qRT-PCR

| **Genes** | **Forward primer** | **Reverse primer** |
| --- | --- | --- |
| FoxO3a | TGGCAAGCACAGAGTTGGATGAAG | CATATCAGTCAGCCGTGGCAGTTC |
| Bim | TCCCACTTCCACCAGCACCATAG | GCACTCATAAGGAGCAGGCACAG |
| c-Myc | TCCTCCCCACGGGCCAGCC | GGCAGGGGTTTGCCTCTTCT |
| p21^Cip1^ | CCACTTTGCCAGCAGAATAA | ACGGGACCGAAGAGACAAC |
| p27^Kip1^ | TCTTCTTCGTCAGCCTCCCTTCC | GTCGCAGAGCCGTGAGCAAG |
| NLRP3 | CAATGGGGAGGAGAAGGCGT | TCTGAACCCCACTTCGGCTC |
| GAPDH | GCACCGTCAAGGCTGAGAAC | GTGGTGAAGACGCCAGTGGA |

**Supplementary Fig. S1.**

**
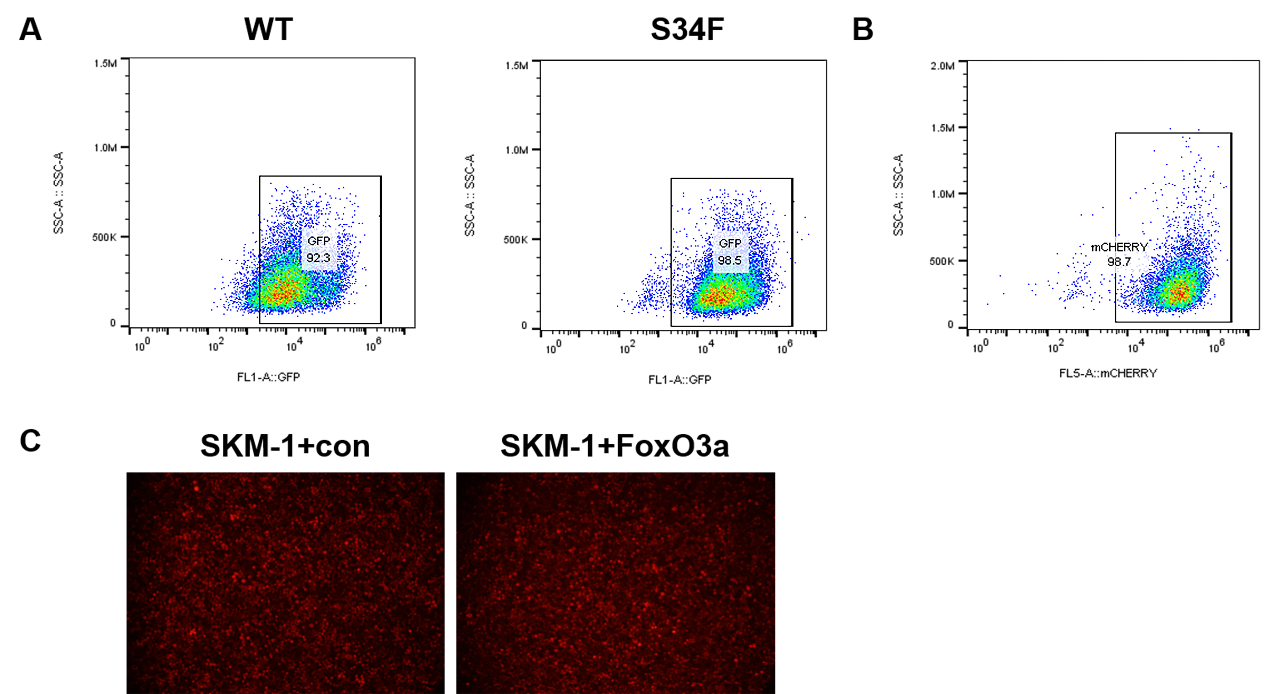
**
